# Associations of objective and perceived social status with well-being in dyads of people with dementia and their caregivers: findings from the IDEAL programme

**DOI:** 10.1007/s00127-025-02933-0

**Published:** 2025-06-05

**Authors:** Yu-Tzu Wu, Laura D. Gamble, Ian Rees Jones, Anthony Martyr, Linda Clare, Fiona E. Matthews

**Affiliations:** 1 Population Health Sciences Institute, Newcastle University, Newcastle upon Tyne, UK; 2 Wales Institute of Social and Economic Research and Data, Cardiff University, Cardiff, UK; 3 University of Exeter Medical School, University of Exeter, Exeter, UK; 4 NIHR Applied Research Collaboration South-West Peninsula, Exeter, UK; 5 Institute for Clinical and Applied Health Research, University of Hull, Hull, UK

**Keywords:** Alzheimer’s disease, APIM, Dyadic analysis, Socioeconomic status, Social standing

## Abstract

**Purpose:**

Social status is related to disparities in health and well-being outcomes in people with dementia (PwD). Few studies have explored the interpersonal influence of social status of PwD on the well-being of their caregiver, or vice versa. We investigated this relationship using measures of objective and perceived (subjective) social status.

**Methods:**

The actor-partner interdependence model was used to investigate dyadic relationships of social status and well-being in 1042 PwD and their spousal caregivers from the IDEAL study. Objective indicators of social status included education, social class and socioeconomic classification. Perceived social status included social comparison and ratings of status in society and in one’s community.

**Results:**

Of the objective social status indicators, actor effects were only observed for caregiver education and their own well-being. Actor effects for perceived social status were stronger and independent of objective social status for both PwD and caregivers. Caregiver social status also influenced the well-being of PwD.

**Conclusion:**

This study provides empirical evidence on the interpersonal influence of social status, especially perceived social status, on well-being in PwD and caregivers. Interactions between PwD, caregivers and wider society may influence the perception of relative social position and impact on living well with dementia.

**Supplementary Information:**

The online version contains supplementary material available at 10.1007/s00127-025-02933-0.

## Introduction

Health inequality across socioeconomic groups has been recognised as an important issue in public health research and policy planning [1]. Indicators of socioeconomic status, such as education and social class, have been widely related to individual health, quality of life, and well-being outcomes in the general population [2–4] as well as in the population living with chronic conditions such as dementia [5–7]. People living with dementia (PwD) and their caregivers often face unequal barriers to accessing care and support [8]. Built on the Determinants of Health model [9], a recent theoretical model for inequalities in dementia has been developed to provide an overview of areas where PwD and their caregivers can experience difficulties and identified key factors in three layers: individual; social & community networks; and society & infrastructure [8]. At the individual level, objective socioeconomic factors are recognised as important factors which can influence awareness of health and social services, access to care and support, and knowledge of dementia management [10, 11]. This may contribute to observed differences in quality of life in PwD and caregivers across objective socioeconomic groups [12].

Whilst studies have found a positive relationship between metrics of objective social status and health and well-being related factors, they have only shown low to modest correlations with personal well-being indicators [13, 14]. Meanwhile, perceived social status, focusing on self-perceived relative standing in the social hierarchy, has been associated with mortality and with physical and psychological health in the general population, and is a powerful predictor of subjective well-being independent of objective social status indicators [15–17].

Less is known about the effects of perceived social status on living well indicators in PwD and their caregivers. Using data from the Improving the experience of Dementia and Enhancing Active Life (IDEAL) study, we have previously reported a positive association between perceived social status and living well indicators (quality of life, life satisfaction, and well-being) in both PwD and in caregivers of PwD [18, 19]. The perception of relative social status might change due to decline in health, restricted social networks, and reduction in income, and might therefore have a different impact on living well outcomes than objective socioeconomic status measures which are often determined at earlier life stages.

Over the last decade, theoretical frameworks of dyadic coping have been developed to consider the adult-care partner dyad in the context of illness and move beyond the traditional individual-based approaches [20]. Built on the Systemic-Transactional Model which suggests that one partner’s stress experiences affect both partners in a committed relationship [21], the Developmental-Contextual Model focuses on chronic illness, a stressful event in the couple’s relationship, and suggests a transactional process of dyadic appraisal, coping strategies, and adjustment over time [22]. Dyadic coping can vary across time (e.g., due to disease stage, age) and be shaped by sociocultural context (e.g., gender roles, access to healthcare) and proximal factors (e.g., quality of marital relationship) [22]. The Theory of Dyadic Illness Management also suggests illness management as a dyadic phenomenon, including three interrelated elements of dyadic appraisal, dyadic management behaviours, and dyadic health [20]. The dyadic appraisal and management behaviours, which can be affected by risk and protective contextual factors (e.g., demographics, culture), influence both care recipient and caregiver health (i.e., dyadic health) and their health also has a feedback loop to the appraisal and behaviours [20].

Interpersonal influences can be particularly important in PwD and their caregivers [23–25]. Due to changes in cognitive and functional ability and challenges of managing daily life, PwD need long-term support and assistance from their caregivers, who are often family members, typically spouses. Spouses and family members are likely to share socioeconomic resources and influence each other’s lifestyle, values, and beliefs. Based on the Theory of Dyadic Illness Management, objective socioeconomic factors and perceived social status may shape care values and preferences in planning and decision making (dyadic appraisal) as well as approaches undertaken to manage the symptoms and seek support from charities, and health and social care providers (dyadic management behaviours). Thus, both objective and perceived social status may influence health and well-being of PwD and their caregivers, with a consequent impact on inequalities in dementia care and support.

Built on our previous IDEAL work [18, 19], this study was based on a large community-based cohort study which included 1042 spousal dyads of PwD and their caregivers across England, Scotland, and Wales. The aim of this study is to investigate the dyadic relationships between objective and perceived social status and well-being and to determine whether the social status of one partner might influence well-being in both that individual and the other member of the dyad.

## Materials and methods

### Study population

The IDEAL study was a longitudinal cohort study of community-dwelling PwD and their caregivers in Great Britain. The study protocol has been published elsewhere [26]. In brief, the IDEAL study was designed to investigate social, psychological, and economic factors that support people living well with dementia. The study population was recruited through a network of 29 National Health Service (NHS) sites across England, Scotland, and Wales between June 2014 and August 2016. Inclusion criteria for participants were a clinical diagnosis of dementia and a Mini-Mental State Examination (MMSE) score of 15 or above. Primary caregivers for the participants were also recruited where possible. These were unpaid family members and friends providing informal care. For those who consented to take part, researchers visited participants to conduct structured interviews and asked caregivers to complete self-reported questionnaires. Written informed consent was secured for all participants. The IDEAL study was approved by the Wales Research Ethics Committee 5 (reference: 13/WA/0405) and the Ethics Committee of the School of Psychology, Bangor University (reference 2014–11684) and was registered with UK Clinical Research Network (UKCRN), registration number 16593. Sample size of the IDEAL study was determined based on findings from the Memory Impairment and Dementia Awareness Study [27] and Dependence in Alzheimer’s Disease in England study [28] to ensure reliability of coefficients based on a proposed analysis using structural equation modelling [29]. Among the 1537 PwD at baseline, 1277 had a caregiver taking part in the study. Since objective social status is likely to have an influence among spouses/partners, the main analysis focused on the 1042 spouse/partner dyads of PwD and caregivers (hereafter referred to as ‘spouse’ caregivers). A sensitivity analysis was conducted comprising all 1277 dyads which includes an additional 235 dyads involving non-spousal family or friend caregivers (hereafter referred to as ‘family’ caregivers). Supplementary Table S1 reports descriptive information about the study population of the 1277 dyads. More than 80% of the caregivers were spouses.

## Measurements

For all PwD and caregivers, self-rated well-being was measured using the percentage score for the World Health Organization-Five Well-being Index (WHO-5) with a range between 0 and 100 [30]. An example item is ‘I have felt cheerful and in good spirits’. Further details on the WHO-5 can be found in Supplementary Materials. The analysis included three measures for objective social status and three measures for perceived social status. Objective social status was measured using the highest educational qualification attained, Registrar General’s Social Class (SC), and the National Statistics Socioeconomic Classification (NS-SEC). Information on the highest educational qualification attained was provided by participants and divided into three groups: low (no qualification), middle (school leaving certificate at age 16), and high (school leaving certificate at age 18 or above). SC and NS-SEC were derived using self-reported information on main occupation and categorized based on the standard occupational classification for the UK (SOC 2010). Although both measures are occupation-based, NS-SEC is specifically designed to measure employment relations, i.e., aspects of work and market situations and of the labour contract [31]. While NS-SEC is recommended to replace SC in recent national statistics, the historical SC scale focused on measuring the hierarchy of occupations according to their reputed standing within UK society [32]. These two measures are designed to capture different aspects of socioeconomic status. Due to limitation of statistical power, the original six levels of SC were combined into three groups: high (I/II), middle (III-NM/III-M) and low (IV/V/VI). Three NS-SEC levels were used in this analysis: high (higher managerial, administrative, and professional occupations), middle (intermediate occupations), and low (routine and manual occupations). For those who had never been employed or did not provide complete information for SOC 2010 coding, social class and NS-SEC were considered as missing data.

Three measures were used to indicate perceived social status in PwD and caregivers. Age-based comparison of social status was measured using one question: ‘Do you think that compared to most other people your age, your overall situation is’ with five levels ‘much worse, somewhat worse, about the same, somewhat better, much better’. PwD and caregivers were asked to rate their perceived social status using the MacArthur Scale of Subjective Social Status [33, 34], which presents images of two ladders. One represents standing in society as a whole and the other represents standing in one’s local community, and participants are asked to place themselves at the appropriate level on each ladder using a scale ranging from 1 (low) to 10 (high). Since the three measures of perceived social status showed potential dose-response relationships with well-being, these were used as ordinal variables in the dyadic analysis.

Covariates included age, sex, dementia type, number of health conditions, cognition, number of hours spent caregiving per day, kin relationship, and co-residence between the person with dementia and caregiver. Age was divided into five groups: <65, 65–69, 70–74, 75–79 and *≥* 80. Dementia types were Alzheimer’s disease (AD), vascular dementia (VaD), mixed AD and VaD, frontotemporal dementia, Parkinson’s disease dementia, dementia with Lewy bodies and other/unspecified. Number of chronic health conditions was assessed with the Charlson Co-morbidity Index [35]. Cognition was assessed using the Mini-Mental State Examination [36]. The relationships between PwD and caregivers (kin relationship) were categorised into two types: spouse/partner and other family/friend. Hours spent caregiving per day was categorised into < 10, 1–10 and 10+.

## Analytical strategy

**Fig. 1:**
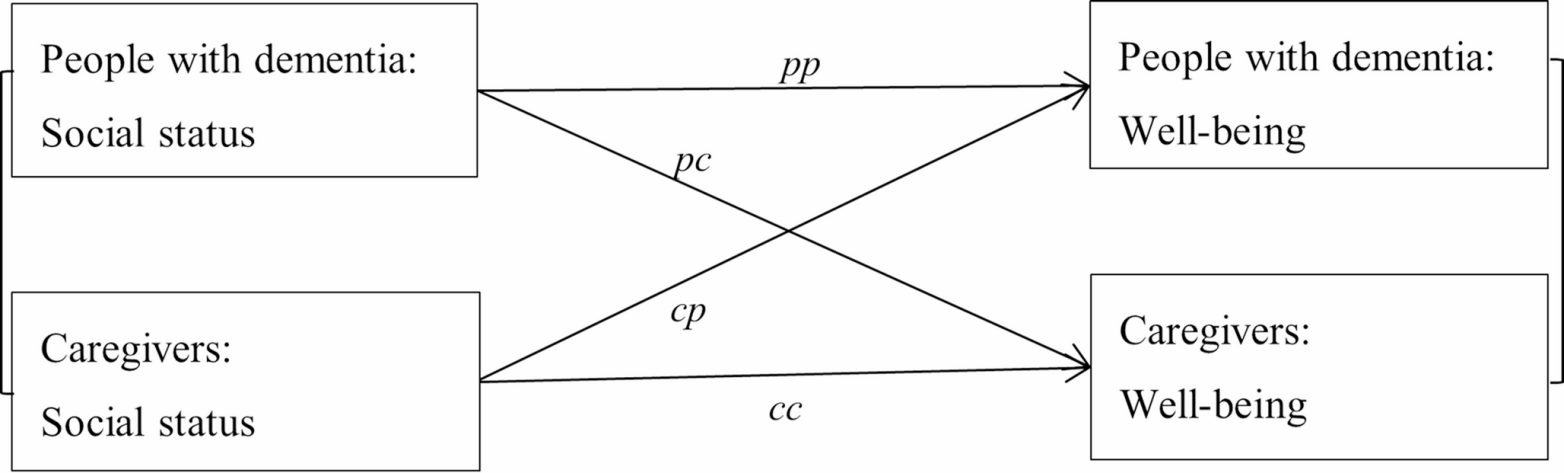
Dyadic relationships between objective and perceived social status and well-being in people with dementia and their caregivers. *pp*: actor effect of social status indicator in PwD on their own well-being; *pc*: partner effect of social status indicator in PwD on well-being in caregivers; *cp*: partner effect of social status indicator in caregivers on well-being in PwD; *cc*: actor effect of social status indicator in caregivers on their own well-being.

Before dyadic analysis, the associations between individual social status measures and well-being in PwD and caregivers were investigated using regression modelling. PwD and caregivers are distinguishable dyad members who need to be considered as two different participants. In the case of distinguishable dyad members, structural equation modelling (SEM) is a straightforward method to fit the actor-partner interdependence model (APIM) [37]. Thus, this study used SEM to investigate the dyadic relationships between objective and perceived social status and well-being in PwD and spouse caregivers (*N* = 1042), adjusting for dementia type, age and sex of PwD and caregivers, number of health conditions, cognition, and hours spent caregiving (Fig. 1). The model estimated four associations including two actor effects of individual social status on individual well-being (*pp* and *cc*) and two partner effects of individual social status on the other’s well-being (*pc* and *cp*). Levels of social status and well-being measures between PwD and spouse caregivers were estimated to account for the dyadic structure. To compare the associations for perceived and objective social status, the analyses on perceived social status were next adjusted for the three objective measures. A further model investigated whether the associations were driven by sex. The dyadic models were stratified by two groups: male PwD– female spouse caregivers (*N* = 686) and female PwD– male spouse caregivers (*N* = 348). A sensitivity analysis was conducted on all dyads (family caregivers, *N* = 1277). We have previously considered an effect size > 5 to be clinically relevant for well-being [38]. To account for missing data, multiple imputation was carried out to generate 20 imputed datasets using all variables in the modelling. Estimates from the imputed datasets were combined according to Rubin’s rules. This study was based on the IDEAL baseline data version 7. All analyses were conducted using Stata 16.

## Results

Descriptive data for the 1042 spousal dyads are reported in Table 1. The mean age was 75 (standard deviation (SD) = 7.8) for PwD and 72 (SD = 8.3) for caregivers. Two thirds of PwD were men and two thirds of caregivers were women, and the most frequent dementia type was Alzheimer’s disease (AD; 56%). Over half of PwD and caregivers had a school leaving certificate at age 18 or above. More than 40% of PwD and caregivers had high levels of SC or NS-SEC, although there were fewer (12%) in the low group for SC than in the low group for NS-Sect. (27%). For perceived social status, PwD and caregivers had similar distributions across the three measures. PwD generally reported higher WHO-5 scores (mean = 61.9; SD = 20.6) than their caregivers (mean = 54.8; SD = 19.9).

There was a weak correlation of WHO-5 scores between PwD and spouse caregivers (Spearman correlation coefficient = 0.21). Among the 1042 dyads, approximately half of the PwD and spouse caregivers had the same levels of education (49%), SC (50%) and NS-SEC (46%). For perceived social status, Spearman correlation coefficients between PwD and spouse caregivers were 0.18 for social comparison, 0.36 for societal ladders, and 0.25 for community ladders. As shown in Supplementary Table S2, there were associations between each objective and perceived social status measure and well-being for both PwD and caregivers in the unadjusted model. In the adjusted model, the associations with well-being only remained for perceived social status measures and caregiver education.

**Table 1:** Descriptive information on the 1042 spousal dyads of people with dementia and their caregivers

Measure		PwD *N* (%)	Spouse caregiver *N* (%)
Age	< 65	95 (9.1)	158 (15.2)
	65–69	147 (14.1)	199 (19.1)
	70–74	207 (19.9)	258 (24.8)
	75–79	265 (25.4)	217 (20.8)
	80+	328 (31.5)	210 (20.2)
Sex	Men	687 (65.9)	349 (33.5)
	Women	355 (34.1)	693 (66.5)
Dementia type	Alzheimer’s disease (AD)	583 (56.0)	–
	Vascular dementia (VaD)	107 (10.3)	–
	Mixed AD & VaD	199 (19.1)	–
	Frontotemporal dementia	43 (4.1)	–
	Parkinson’s disease dementia	40 (3.8)	–
	Dementia with Lewy bodies	39 (3.7)	–
	Other/Unspecified	31 (3.0)	–
Education	Low	252 (24.3)	256 (24.7)
	Middle	189 (18.2)	233 (22.5)
	High	596 (57.5)	549 (52.9)
	Missing	5	4
Hours spent caregiving per day	< 10	–	207 (20.1)
	1–10	–	371 (36.1)
	10+	–	450 (43.8)
	Missing	–	14
Social class	Low	124 (12.1)	88 (8.7)
	Middle	434 (42.4)	435 (43.1)
	High	465 (45.5)	487 (48.2)
	Missing	19	32
NS-SEC	Low	278 (27.2)	231 (22.9)
	Middle	295 (28.8)	353 (35.0)
	High	450 (44.0)	426 (42.2)
	Missing	19	32
Social comparison	Median (IQR)	3.0 (1.0)	3.0 (1.0)
	Missing	39	15
Societal ladder	Median (IQR)	7.0 (2.0)	7.0 (2.0)
	Missing	20	42
Community ladder	Median (IQR)	6.0 (3.0)	6.0 (2.0)
	Missing	54	54
Number of health conditions	Median (IQR)	1.0 (2.0)	1.0 (2.0)
	Missing	34	72
Cognition (MMSE)	Median (IQR)	23.0 (6.0)	–
	Missing	1	–
Well-being (WHO-5)	Median (IQR)	64.0 (24.0)	56.0 (32.0)
	Missing	19	28

*Notes*:NS-SEC, National Statistics Socio-Economic Classification; MMSE, Mini-Mental State Examination; WHO-5, World Health Organization-Five Well-being Index; PwD, people with dementia; IQR, interquartile range

Table 2 reports the dyadic associations between objective social status measures and well-being in PwD and caregivers following adjustment for age, sex, dementia type, number of health conditions, cognition, and number of hours spent caregiving per day. Actor effects were only found for caregiver education and caregiver well-being (*cc*); caregivers with low education (−4.94; 95% CI: −7.84, −2.03) reported lower WHO-5 scores compared to their counterparts with higher objective social status. For the PwD, there was limited evidence of actor effects (*pp*) for education, SC and NS-SEC. In terms of partner effects (*pc* and *cp*), PwD whose caregivers had low education (−3.31, 95% CI: −6.33, −0.31) reported lower well-being (*cp*) compared to their PwD counterparts whose caregivers had higher education. There was limited evidence of partner effects of PwD education, SC or NS-SEC on caregiver well-being (*pc*).

**Table 2:** The dyadic relationships between objective social status and well-being in people with dementia and their spouse caregivers

Measures	Person with dementia social status		Caregiver social status	
	pp	pc	cc	cp
	Estimate (95% CI)	Estimate (95% CI)	Estimate (95% CI)	Estimate (95% CI)
Education				
High	(ref.)	(ref.)	(ref.)	(ref.)
Middle	1.10 (−2.12, 4.33)	−0.04 (−3.15, 3.07)	−2.66 (−5.56, 0.23)	−0.73 (−3.75, 2.28)
Low	−1.11 (−4.16, 1.94)	−0.36 (−3.32, 2.59)	−4.94 (−7.84, −2.03)	−3.31 (−6.33, −0.31)
Social class				
High	(ref.)	(ref.)	(ref.)	(ref.)
Middle	0.69 (−1.99, 3.36)	−1.12 (−3.65, 1.41)	−1.85 (−4.34, 0.63)	−1.46 (−4.08, 1.16)
Low	−3.04 (−7.10, 1.03)	−0.78 (−4.60, 3.04)	−3.47 (−7.85, 0.91)	−3.43 (−7.99, 1.12)
NS-SEC				
High	(ref.)	(ref.)	(ref.)	(ref.)
Middle	0.22 (−2.75, 3.19)	−1.20 (−4.05, 1.66)	−0.50 (−3.24, 2.23)	−0.78 (−3.62, 2.07)
Low	−0.52 (−3.67, 2.63)	−1.00 (−3.98, 1.97)	−2.48 (−5.68, 0.72)	−3.08 (−6.36, 0.21)

*Notes*: NS-SEC, National Statistics Socio-Economic Classification; CI, confidence intervals; ref, reference category. *pp*, *pc*, *cc* and *cp* refer to the four dyadic pathways shown in Fig. 1. Models were adjusted for age, sex, dementia type, number of health conditions, cognition, and hours spent caregiving per day

Table 3 reports the results of perceived social status. Actor effects for both PwD and spouse caregivers (*pp* and *cc*) were found for all three measures of perceived social status (Table 3; adjusted 1). For PwD, higher WHO-5 scores were found in those who reported high levels of social comparison (5.69; 95% CI: 4.51, 6.87) and made higher ratings on the societal (2.24; 95% CI: 1.49, 2.99) and community ladders (2.09; 95% CI: 1.46, 2.72). For caregivers, higher well-being scores were found in those who reported higher levels of social comparison (6.00; 95% CI: 4.73, 7.27) and made higher ratings on the societal (2.57; 95% CI: 1.71, 3.44) and community ladders (2.21; 95% CI: 1.53, 2.90). There were partner effects for the caregiver’s perceived social status and the well-being of the PwD (*cp*), although they were weaker than the actor effects; higher levels of social comparison (2.21; 95% CI: 0.94, 3.48) and higher ratings on the societal ladder (0.89; 95% CI: 0.01, 1.78) and community ladders (0.84; 95% CI: 0.13, 1.56) in caregivers were associated with higher well-being scores in PwD. There was limited evidence of partner effects of PwD perceived social status on caregiver well-being (*pc*). Findings were upheld when each model was adjusted for objective social indicators (Table 3; adjusted 2).

**Table 3:** The dyadic relationships between perceived social status and well-being in people with dementia and spouse caregivers

Measures	Person with dementia social status		Caregiver social status	
	pp	pc	cc	cp
	Estimate (95% CI)	Estimate (95% CI)	Estimate (95% CI)	Estimate (95% CI)
Higher social comparison				
Adjusted 1	5.69 (4.51, 6.87)	0.22 (−0.96, 1.40)	6.00 (4.73, 7.27)	2.21 (0.94, 3.48)
Adjusted 2	5.61 (4.43, 6.79)	0.20 (−0.97, 1.38)	5.95 (4.69, 7.22)	2.25 (0.97, 3.52)
Higher societal ladder				
Adjusted 1	2.24 (1.49, 2.99)	0.51 (−0.21, 1.23)	2.57 (1.71, 3.44)	0.89 (0.01, 1.78)
Adjusted 2	2.29 (1.53, 3.05)	0.44 (−0.28, 1.17)	2.43 (1.54, 3.33)	0.96 (0.03, 1.90)
Higher community ladder				
Adjusted 1	2.09 (1.46, 2.72)	0.44 (−0.17, 1.06)	2.21 (1.53, 2.90)	0.84 (0.13, 1.56)
Adjusted 2	2.07 (1.44, 2.70)	0.38 (−0.24, 1.00)	2.10 (1.40, 2.81)	0.84 (0.11, 1.57)

*Notes*: CI, confidence intervals; ref, reference category. *pp*, *pc*, *cc* and *cp* refer to the dyadic pathways shown in Fig. 1. Adjusted 1: adjusted for dementia types, age, sex, number of health conditions, cognition, and hours spent caregiving per day; Adjusted 2: additionally adjusted for PwD and caregiver education, social class and NS-SEC

When all measures of objective socioeconomic status and perceived social status were included in one model (Supplementary Material 1; Figure S1), actor effects remained for perceived social status indicators and caregiver education, but partner effects only remained for spousal caregiver social comparison and PwD well-being (Supplementary Material 1; Table S3). A sensitivity analysis was conducted incorporating all dyads (those with both spouse and family/friend caregivers) and findings were similar to those reported for spouse only dyads (Supplementary Material 2; Tables S4-S6).

Since socioeconomic status of married couples was historically determined by the occupation of the husband, within the couples we investigated whether the associations were driven by sex. Analysis stratified by sex composition of the dyadic couples suggested that some of the dyadic associations may be modified by the sex composition of couples. However, these analyses lacked statistical power. More detailed information is provided in Supplementary Material 3, Tables S7-S9.

## Discussion

This study investigated relationships between indicators of objective social status, perceived social status, and subjective well-being in dyads using a large cohort study of PwD and their spousal caregivers. In the dyadic model, no objective social status indicators of the PwD were associated with their own well-being and only caregiver education was associated with caregiver well-being. Perceived measures of social status in both PwD and caregivers were strongly associated with their own well-being and this was upheld when controlling for objective social status. Partner effects were observed for caregiver education and caregiver perceived social status measures with well-being of the PwD, but neither objective nor perceived social status of the PwD were associated with the well-being of the caregiver.

Several studies have shown that perceived social status is a powerful predictor of subjective well-being in the general population, and this is independent of objective social class indicators [16, 39, 40]. This is in agreement with the findings for both PwD and caregivers in the present study, and in our previous studies in PwD and caregivers using IDEAL data [18, 19]. This indicates that the perception of relative standing in society and in local communities is more important than those socioeconomic determinants in earlier life, and suggests a psychosocial pathway by which interactions between PwD, caregivers and wider society might influence psychological health and well-being [41]. Individuals who perceive themselves as having a high rank in society tend to have a stronger sense of being able to control and influence their social environment, whereas individuals with a low perceived rank experience social constraints, helplessness, and uncertainty [42].

Interestingly, we found that not only do education and perceived social status of caregivers influence caregivers’ own well-being, but they also influence the well-being of the PwD, However, these social status measures of PwD did not impact on caregiver well-being. The partner relationship suggests a potential interpersonal influence. A diagnosis of dementia changes the pre-existing relationship of a spouse couple, with one member taking on the role of caregiver and the other becoming more dependent on the caregiver [43]. Unlike objective social status which focuses on socioeconomic positions of individuals, perceived social status is likely to reflect conditions of a person’s upbringing, with lasting impact on their personal and social identities as well as their reactions, response, and behaviours to events in their life [42]. Thus, perceived social status of caregivers, together with their education, might be more likely to influence their values and preferences in dementia care. Based on an example of the Theory of Dyadic Illness Management, PwD and caregivers who have shared views on how to move forward with the care planning process results in a care plan that is better aligned with the wishes of the PwD, and empowers the caregiver to make decisions when needed [20]. On the other hand, if caregivers are in full charge of care planning and decision making and their views are not aligned with those of the PwD, these inconsistent views may lead to tension and stress and have detrimental impacts on health and well-being of the dyad. Caregivers might play a more important role in this dyadic relationship as they will eventually lead care planning and management for PwD when dementia is at a more advanced stage. This may explain why the partner effect was only observed in caregivers, not PwD. In addition, caregivers’ ratings of perceived social status might reflect their stress levels and their experiences of taking care of the person with dementia [44, 45]. There is evidence from previous studies that the caregiver’s experience influences the ability of the person with dementia to live well, with higher caregiving burden, higher stress, and higher perceived social restrictions being associated with lower self-rated measures of living well for the person with dementia [12, 46–50]. Caregivers who reported higher levels of perceived social status may have greater control over their daily life and have more supportive networks to facilitate caregiving, leading to better mood and improved well-being. This may enable them to provide appropriate support for the person with dementia, which in turn impacts positively on the well-being of the person with dementia.

For objective measures of social status, we identified limited actor effects in the dyadic model, finding an association only between caregiver education and caregiver well-being. Other studies have found that the associations between objective social status indicators and health and well-being outcomes are present but are weaker than perceived social status measures [13, 14, 17]. We have previously found some association between education and social class and capability to live well in PwD and their caregivers in the IDEAL cohort [18, 51]. However, whilst we do see some effects in the unadjusted models, these effects are lost when cognition of the PwD, health conditions of the PwD and caregiver, and number of hours spent caregiving per day are controlled for, suggesting they are not robust. The relationship between caregiver education and caregiver well-being may result from higher education being linked with better health literacy, which is defined as the combination of personal competencies and situational resources needed for people to access, understand, appraise, and use information and services to make decisions about health [52], to manage their care duties, and seek the support needed from health and social care professionals, community-based organizations and charities [53, 54]. For example, in the UK, social care needs assessment is essential for PwD and caregivers to receive subsidised care but awareness of and access to the assessment have been identified as key barriers to dementia care [10]. Caregivers’ education might play an important role in navigating the complicated process and receiving subsidised care, which can support PwD, their care planning and management with subsequent influences on their well-being. The lack of actor effects for social class and socioeconomic status in caregivers could be influenced by gender roles and societal expectations in current cohorts of older people, particularly women. Historically, socioeconomic status of a couple was determined by the husband’s occupation and thus a wife’s social status according to their occupation may not align with their level of education.

## Strengths and limitations

The IDEAL study recruited a large number of community-dwelling PwD and caregivers in England, Scotland, and Wales and collected a wide range of data on social, psychological, and physical health factors. This study assessed social status through both objective and perceived measures, including not only general socioeconomic indicators based on education and occupation but also perceived social status reported by PwD and caregivers. In particular, the assessment of perceived status included an age-based comparison and self-ratings for status in both society and the community, which has seldom been done in previous investigations [33]. The analysis included information from PwD and caregivers and used advanced modelling methods to investigate inter-relationships in the dyads.

While the method of dyadic analysis can be an effective approach to explore the interpersonal influences of different psychosocial factors on health and well-being outcomes in PwD and caregivers, the present cross-sectional study was not able to address reverse causality. It is possible that individual well-being might influence ratings of perceived social status. Many IDEAL participants and caregivers had a relatively high educational level and socioeconomic status. People with low objective socioeconomic status might be under-represented in this study population. Measures for social class and socioeconomic classification were based on the main occupation in working life and some participants had never been employed, although the number in this group was generally small (< 5%). Nevertheless, this risks skewing the class experiences of women, especially those who were not in formal employment or were in part-time employment as they were likely responsible for household and child care. Whilst the indices of objective social status included in the study would remain the same throughout an older person’s life, other measures such as income might not. Income was a poorly answered question in IDEAL so could not be studied. Those with advanced dementia were not recruited to the IDEAL study but the dyadic relationships between social status and well-being might be different when dementia has progressed to more advanced stages. However, it may not be appropriate or possible to ask people with advanced dementia to rate social status and respond to complicated questions. Due to limited statistical power, measures for educational levels and social class were combined into three groups. However, this did not affect the potential for examining trends from low to high levels of socioeconomic status. Since measures for objective and perceived social status as well as dementia care can vary across different countries, cultures, and societies, the results of the present study may not be generalizable to PwD and caregivers outside of the UK.

## Conclusions

This study offers a more nuanced and multidimensional understanding of the impact of inequalities, both structurally (objective positioning) and psychologically (perceptions of status). The findings suggest that perceived social status may be more important than objective social status in supporting well-being in PwD and caregivers. Compared to objective socioeconomic status factors such as education and social class, the perception of social status is potentially modifiable and could be an indictor which reflects individual psychological status (e.g., dementia-related stress level), caregiving experience, or interactions with spouses, family, local communities and wider society. It is important to identify factors contributing to poor ratings of perceived social status so that interventions can be developed to support people and their caregivers to live well with dementia. Given that caregivers’ perceived social status influences both their own well-being and that of the person with dementia, this study suggested that more support should be directed at informal caregivers. If their perceptions of standing in society could be improved, then living well outcomes could be better for both them and the care recipient and may potentially keep them in their caregiving role for longer. Targeted support for caregivers may offer a route to helping improve both caregiver and PwD well-being.

## Electronic supplementary material

Below is the link to the electronic supplementary material.

Supplementary Material file1 (DOCX 108 KB)

## Data Availability

IDEAL data were deposited with the UK data archive in April 2020. Details of how the data can be accessed can be found here: http://reshare.ukdataservice.ac.uk/854293/.
