# Supplementary material for: Associations of objective and perceived social status with well-being in dyads of people with dementia and their caregivers: findings from the IDEAL programme
Source: Soc Psychiatry Psychiatr Epidemiol. 2025 Jun 5;61(6):1071–80. doi: 10.1007/s00127-025-02933-0 (PMC13226339; doi:10.1007/s00127-025-02933-0)
Supplement: Supplementary file 1 — Supplementary Material file1 (DOCX 108 KB) [file 127_2025_2933_MOESM1_ESM.docx]

Associations of objective and perceived social status with well-being in dyads of people with dementia and their caregivers: findings from the IDEAL programme

**Supplementary Materials**

**Measurement of well-being**

World Health Organization-Five Well-being Index (WHO-5) is a self-report instrument measuring mental well-being. It consists of 5 statements, each of which are rated on a 6-point Likert scale ranging from ‘all the time’ (5) to ‘at no time’ (0):

- I have felt cheerful and in good spirits
- I have felt calm and relaxed
- I have felt active and vigorous
- I woke up feeling fresh and rested
- My daily life has been filled with things that interest me.

The total score is calculated (ranging from 0-25) and multiplied by 4 to convert to a percentage.

**Table S1: Descriptive information on the population of 1277 dyads of people with dementia and family caregivers in the IDEAL study**

| Measure | | People with dementia  N (%) | | Family caregivers  N (%) |
| --- | --- | --- | --- | --- |
| Age | <65 | 102 (8.0) | | 365 (28.6) |
|  | 65-69 | 159 (12.5) | | 209 (16.4) |
|  | 70-74 | 230 (18.0) | | 265 (20.8) |
|  | 75-79 | 303 (23.7) | | 224 (17.5) |
|  | 80+ | 483 (37.8) | | 214 (16.8) |
| Sex | Men | 750 (58.7) | | 394 (30.9) |
|  | Women | 527 (41.3) | | 883 (69.1) |
| Dementia type | Alzheimer’s disease (AD) | 712 (55.8) | | - |
|  | Vascular dementia (VaD) | 141 (11.0) | | - |
|  | Mixed AD & VaD | 261 (20.4) | | - |
|  | Frontotemporal dementia | 45 (3.5) | | - |
|  | Parkinson’s disease dementia | 43 (3.4) | | - |
|  | Dementia with Lewy bodies | 43 (3.4) | | - |
|  | Other/Unspecified | 32 (2.5) | | - |
| Type of relationship | Spouse/partner | - | | 1042 (81.6) |
|  | Family/friend | - | | 235 (18.4) |
| Hours spent caregiving per day | <10 | - | | 277 (22.0) |
|  | 1-10 | - | | 498 (39.5) |
|  | 10+ | - | | 487 (38.6) |
|  | Missing | - | | 15 |
| Co-resident PwD/caregiver | Yes | - | | 1057 (83.8) |
|  | No | - | | 205 (16.2) |
|  | Missing | - | | 15 |
| Education | Low | 348 (27.3) | | 271 (21.4) |
|  | Middle | 227 (17.8) | | 284 (22.4) |
|  | High | 697 (54.6) | | 714 (56.3) |
|  | missing | 5 | | 8 |
| Social class | Low | 159 (12.7) | | 110 (8.9) |
|  | Middle | 539 (43.1) | | 524 (42.5) |
|  | High | 554 (44.3) | | 599 (48.6) |
|  | missing | 25 | | 44 |
| NS-SEC | Low | 351 (28.0) | | 268 (21.7) |
|  | Middle | 377 (30.1) | | 441 (35.8) |
|  | High | 524 (41.9) | | 524 (42.5) |
|  | missing | 25 | | 44 |
| Social comparison | Median (IQR) | 4.0 (1.0) | | 3.0 (1.0) |
|  | missing | 46 | | 21 |
| Societal ladder | Median (IQR) | 7.0 (2.0) | | 7.0 (2.0) |
|  | missing | 29 | | 51 |
| Community ladder | Median (IQR) | 6.0 (3.0) | | 6.0 (2.0) |
|  | missing | 78 | | 68 |
| Number of health conditions | Median (IQR) | | 1.0 (2.0) | 1.0 (2.0) |
|  | missing | | 43 | 90 |
| Cognition (MMSE) | Median (IQR) | | 23.0 (6.0) | - |
|  | missing | | 1 | - |
| Well-being (WHO-5) | Median (IQR) | | 64.0 (28.0) | 56.0 (32.0) |
|  | missing | | 23 | 36 |

*Notes*: NS-SEC, National Statistics Socio-Economic Classification; MMSE, Mini-Mental State Examination; WHO-5, World Health Organization-Five Well-being Index; IQR, interquartile range.

**Table S2. Associations between each measure of objective and perceived social status with well-being of the person with dementia, or with the well-being of the spouse caregiver**

|  | | Univariable model  Estimate (95% CI) | Adjusted model^  Estimate (95% CI) |
| --- | --- | --- | --- |
| *Person with dementia measures* | |  |  |
| Education | High | (ref.) | (ref.) |
|  | Middle | 0.57 (-2.80 – 3.95) | 0.79 (-2.43 – 4.01) |
|  | Low | -2.54 (-5.58 – -0.51) | -1.90 (-4.87 – 1.07) |
| Social class | High | (ref.) | (ref.) |
|  | Middle | -0.84 (-3.55 – 1.87) | 0.22 (-2.36 – 2.79) |
|  | Low | -6.06 (-10.22 - -1.90) | -3.42 (-7.43 – 0.59) |
| NS-SEC | High | (ref.) | (ref.) |
|  | Middle | -1.26 (-4.30 – 1.77) | -0.15 (-3.05 – 2.76) |
|  | Low | -2.93 (-6.02 – 0.17) | -1.26 (-4.27 – 1.74) |
| Higher social comparison | | 7.29 (6.10 – 8.47) | 5.94 (4.77 – 7.12) |
| Higher societal ladder | | 3.06 (2.33 – 3.78) | 2.47 (1.77 – 3.18) |
| Higher community ladder | | 2.63 (1.99 – 3.26) | 2.27 (1.66 – 2.88) |
| *Spouse caregiver measures* | |  |  |
| Education | High | (ref.) | (ref.) |
|  | Middle | -4.15 (-7.18 - -1.11) | -2.72 (-5.60 – 0.17) |
|  | Low | -6.58 (-9.52 - -3.64) | -5.01 (-7.83 - -2.19) |
| Social class | High | (ref.) | (ref.) |
|  | Middle | -2.80 (-5.39 - -0.22) | -2.08 (-4.51 – 0.35) |
|  | Low | 5.31 (-9.81 - -0.80) | -3.81 (-8.10 – 0.48) |
| NS-SEC | High | (ref.) | (ref.) |
|  | Middle | -2.69 (-5.51 – 0.13) | -0.71 (-3.40 – 1.97) |
|  | Low | -4.60 (-7.77 - -1.43) | -2.77 (-5.82 – 0.27) |
| Higher social comparison | | 7.44 (6.28 – 8.61) | 6.02 (4.76 – 7.27) |
| Higher societal ladder | | 3.45 (2.62 – 4.28) | 2.75 (1.93 – 3.57) |
| Higher community ladder | | 2.79 (2.10 – 3.47) | 2.33 (1.67 – 2.99) |

*Notes*: NS-SEC, National Statistics Socio-Economic Classification; CI, confidence intervals; ref, reference category. *^* Person with dementia models were adjusted for age, sex, dementia type, number of health conditions, cognition of the person with dementia. Spouse caregiver models were adjusted for age, sex, and number of health conditions of the caregiver and number of hours spent caregiving per day.

Supplementary Material 1. Objective and perceived social status

The model in Figure S1 included all six measures of perceived and objective social status in people with dementia and spouse caregivers and their dyadic relationships with well-being. The results for all perceived and objective social status measures are reported in Table S3.

**Figure S1: Dyadic relationships between perceived and objective social status and well-being (adjusted for age, sex, dementia type, number of health conditions, cognition, and hours spent caregiving per day)**

PwD: age, sex, dementia type, number of health conditions, cognition

PwD: Perceived

*pp*

*pc*

PwD: WHO-5

*cp*

Caregiver: Perceived

*cc*

*pp*

PwD: Objective

*pc*

Caregiver: WHO-5

*cp*

Caregiver: Objective

*cc*

Caregiver: age, sex, dementia type of PwD, number of health conditions, hours spent caregiving per day

**Table S3: Results of dyadic relationships between perceived and objective social status and well-being in spousal dyads of people with dementia and their caregivers when all measures are included in the same model**

|  | Person with dementia social status | | Spouse caregiver social status | |
| --- | --- | --- | --- | --- |
|  | *pp* | *pc* | *cc* | *cp* |
| Measures | Estimate (95% CI) | Estimate (95% CI) | Estimate (95% CI) | Estimate (95% CI) |
| Perceived |  |  |  |  |
| Higher social comparison | 4.81 (3.61, 6.00) | -0.25 (-1.45, 0.95) | 5.46 (4.19, 6.72) | 1.91 (0.63, 3.19) |
| Higher societal ladder | 1.01 (0.19, 1.83) | 0.44 (-0.36, 1.24) | 0.85 (-0.18, 1.88) | -0.04 (-1.12, 1.04) |
| Higher community ladder | 1.10 (0.42, 1.79) | 0.08 (-0.60, 0.75) | 1.51 (0.72, 2.30) | 0.66 (-0.17, 1.49) |
| Objective |  |  |  |  |
| Education |  |  |  |  |
| High | (ref.) | (ref.) | (ref.) | (ref.) |
| Middle | 2.27 (-0.88, 5.42) | 1.64 (-1.41, 4.69) | -1.56 (-4.50, 1.39) | 0.45 (-2.56, 3.47) |
| Low | 0.64 (-2.46, 3.73) | 0.89 (-2.10, 3.89) | -3.22 (-6.32, -0.12) | -1.71 (-4.85, 1.43) |
| Social class |  |  |  |  |
| High | (ref.) | (ref.) | (ref.) | (ref.) |
| Middle | 0.24 (-3.43, 3.91) | -0.80 (-4.37, 2.77) | 0.70 (-2.91, 4.30) | 0.71 (-2.97, 4.39) |
| Low | -1.68 (-6.46, 3.09) | -0.38 (-5.04, 4.27) | 0.81 (-4.36, 5.97) | 0.46 (-4.99, 5.90) |
| NS-SEC |  |  |  |  |
| High | (ref.) | (ref.) | (ref.) | (ref.) |
| Middle | 0.75 (-2.98, 4.49) | -0.51 (-4.20, 3.18) | 0.97 (-2.58, 4.51) | -0.42 (-4.03, 3.19) |
| Low | 1.55 (-2.79, 5.90) | 1.38 (-2.84, 5.61) | 0.05 (-4.45, 4.55) | -1.68 (-6.28, 3.91) |

*Notes*: NS-SEC, National Statistics Socio-Economic Classification; ref, reference category; CI, confidence intervals. *pp*, *pc*, *cc* and *cp* refer to the dyadic pathways shown in Figure S1. Adjusted for dementia type, age, sex, number of health conditions, cognition, and hours spent caregiving per day.

Supplementary Material 2: Sensitivity analysis incorporating all dyads of people with dementia and family caregivers (N=1277)

**Table S4: Dyadic relationship between objective social status and well-being in people with dementia and their family caregiver (N=1277)**

|  | Person with dementia | | Family caregiver |  |
| --- | --- | --- | --- | --- |
|  | *pp* | *pc* | *cc* | *cp* |
| Measures | Estimate (95% CI) | Estimate (95% CI) | Estimate (95% CI) | Estimate (95% CI) |
| Education |  |  |  |  |
| High | (ref.) | (ref.) | (ref.) | (ref.) |
| Middle | 0.21 (-2.75, 3.20) | -0.20 (-3.04, 2.64) | -3.36 (-5.97, -0.75) | 0.70 (-2.01, 3.40) |
| Low | -1.08 (-3.76, 1.60) | 0.16 (-2.41, 2.73) | -5.15 (-7.92, -3.39) | -3.09 (-5.97, -0.22) |
| Social class |  |  |  |  |
| High | (ref.) | (ref.) | (ref.) | (ref.) |
| Middle | -0.68 (-3.09, 1.74) | -0.65 (-2.94, 1.63) | -1.69 (-3.96, 0.57) | -1.05 (-3.43, 1.33) |
| Low | -3.29 (-6.95, 0.37) | -2.74 (-6.15, 0.66) | -2.77 (-6.63, 1.09) | -1.06 (-5.12, 3.00) |
| NS-SEC |  |  |  |  |
| High | (ref.) | (ref.) | (ref.) | (ref.) |
| Middle | -0.40 (-3.07, 2.26) | -0.41 (-2.96, 2.14) | -0.67 (-3.10, 1.76) | -1.00 (-3.56, 1.56) |
| Low | -1.32 (-4.15, 1.51) | -0.76 (-3.45, 1.93) | -3.18 (-6.08, -0.27) | -2.05 (-5.04, 0.94) |

*Notes*: NS-SEC, National Statistics Socio-Economic Classification; ref, reference category; CI, confidence intervals. *pp*, *pc*, *cc* and *cp* refer to the dyadic pathways shown in Figure 1. Models are adjusted for age, sex, dementia type, number of health conditions, cognition, type of relationship, hours spent caregiving per day and co-resident person with dementia/caregiver.

**Table S5. The dyadic relationships between perceived social status and well-being in people with dementia and family caregivers (N=1277)**

|  | Person with dementia | | Family caregiver |  |
| --- | --- | --- | --- | --- |
|  | *pp* | *pc* | *cc* | *cp* |
| Measures | Estimate (95% CI) | Estimate (95% CI) | Estimate (95% CI) | Estimate (95% CI) |
| Higher social comparison |  |  |  |  |
| Adjusted 1 | 5.71 (4.62, 6.80) | 0.43 (-0.62, 1.48) | 6.47 (5.34, 7.60) | 1.21 (0.07, 2.35) |
| Adjusted 2 | 5.66 (4.57, 6.76) | 0.38 (-0.67, 1.43) | 6.43 (5.30, 7.56) | 1.21 (0.07, 2.36) |
| Higher societal ladder |  |  |  |  |
| Adjusted 1 | 2.24 (1.58, 2.91) | 0.46 (-0.18, 1.10) | 2.72 (1.96, 3.48) | 0.89 (0.11, 1.68) |
| Adjusted 2 | 2.29 (1.61, 2.97) | 0.37 (-0.27, 1.02) | 2.58 (1.80, 3.36) | 0.93 (0.12, 1.74) |
| Higher community ladder |  |  |  |  |
| Adjusted 1 | 2.00 (1.44, 2.57) | 0.38 (-0.18, 0.94) | 2.24 (1.63, 2.85) | 0.83 (0.19, 1.47) |
| Adjusted 2 | 1.99 (1.42, 2.55) | 0.31 (-0.25, 0.87) | 2.13 (1.51, 2.76) | 0.81 (0.16, 1.46) |

*Notes*: CI, confidence intervals; ref, reference category. *pp*, *pc*, *cc* and *cp* refer to the dyadic pathways shown in Figure 1. Adjusted 1: adjusted for dementia type, age and sex, number of health conditions, cognition, hours spent caregiving per day, person with dementia/caregiver co-residence and type of relationship; Adjusted 2: additionally adjusted for education, social class and NS-SEC.

**Table S6: Results for dyadic relationships between perceived and objective social status and well-being in dyads of people with dementia and their family caregivers when all measures of perceived and objective social status are included in the same model**

|  | Person with dementia | | Family caregiver |  |
| --- | --- | --- | --- | --- |
|  | *pp* | *pc* | *cc* | *cp* |
| Measures | Estimate (95% CI) | Estimate (95% CI) | Estimate (95% CI) | Estimate (95% CI) |
| Perceived |  |  |  |  |
| Higher social comparison | 4.83 (3.73, 5.94) | -0.02 (-1.09, 1.04) | 5.86 (4.72, 7.00) | 0.88 (-0.29, 2.04) |
| Higher societal ladder | 1.01 (0.26, 1.76) | 0.39 (-0.33, 1.10) | 0.86 (-0.06, 1.78) | 0.16 (-0.81, 1.13) |
| Higher community ladder | 1.10 (0.48, 1.72) | 0.08 (-0.52, 0.67) | 1.42 (0.71, 2.13) | 0.65 (-0.10, 1.40) |
| Objective |  |  |  |  |
| Education |  |  |  |  |
| High | (ref.) | (ref.) | (ref.) | (ref.) |
| Middle | 1.63 (-1.26, 4.53) | 1.33 (-1.43, 4.09) | -2.11 (-4.71, 0.50) | 2.13 (-0.57, 4.83) |
| Low | 1.19 (-1.60, 3.99) | 1.65 (-0.98, 4.28) | -3.13 (-6.00, -0.26) | -1.32 (-4.31, 1.66) |
| Social class |  |  |  |  |
| High | (ref.) | (ref.) | (ref.) | (ref.) |
| Middle | -1.32 (-4.66, 2.02) | -1.09 (-4.23, 2.06) | 1.41 (-1.81, 4.63) | 0.37 (-3.01, 3.75) |
| Low | -2.14 (-6.59, 2.31) | -2.66 (-6.84, 1.51) | 1.99 (-2.61, 6.59) | 1.62 (-3.32, 6.55) |
| NS-SEC |  |  |  |  |
| High | (ref.) | (ref.) | (ref.) | (ref.) |
| Middle | 1.12 (-2.24, 4.48) | 0.35 (-2.91, 3.61) | 0.44 (-2.74, 3.63) | -0.74 (-4.06, 2.58) |
| Low | 2.17 (-1.81, 6.16) | 1.81 (-2.91, 5.60) | -1.41 (-5.50, 2.68) | -1.29 (-5.55, 2.96) |

*Notes*: NS-SEC, National Statistics Socio-Economic Classification; ref, reference category; CI, confidence intervals. *pp*, *pc*, *cc* and *cp* refer to the dyadic pathways shown in Figure S1. Models are adjusted for dementia type, age and sex, number of health conditions, cognition, hours spent caregiving per day, person with dementia/caregiver co-residence and type of relationship.

Supplementary Material 3. Sex and type of relationships

Of the 1042 dyads where the person with dementia had a spouse or partner caregiver, a small number of same sex couples were excluded (n=8, 0.8%). In total, this analysis included 1034 couples; 686 males living with dementia and their female spouse caregivers, and 348 females living with dementia and their male spouse caregivers. Table S7 reports numbers and percentages of people with dementia and spouse caregivers by the three factors of objective social status, stratified by sex. In general, men had higher education than women.

Table S8 reports unadjusted results of dyadic relationships between objective social status and well-being in the 1034 spouse dyads stratified by the sex composition of the couples: males living with dementia and female caregivers, and females living with dementia and male caregivers. Compared to the dyads of males living with dementia and female caregivers, both actor and partner effects were mostly stronger in the dyads of females living with dementia and male caregivers. In particular, objective social status of male spouse caregivers was associated with well-being in themselves and the female living with dementia. However, the analysis suffered from a lack of statistical power and 95% CIs were wide and overlapped across two groups. Similar results were found for perceived social status (Table S9).

**Table S7: Number and percentage of people with dementia and spouse caregivers by objective social status (N=1034)**

|  | Person with dementia | | Spouse caregiver | |
| --- | --- | --- | --- | --- |
| Measures | Male PwD – Female caregiver | Female PwD – Male caregiver | Male PwD – Female caregiver | Female PwD – Male caregiver |
| Education (N, %) |  |  |  |  |
| High | 448 (65.7) | 145 (41.8) | 329 (48.0) | 215 (62.3) |
| Middle | 101 (14.8) | 84 (24.2) | 176 (25.7) | 55 (15.9) |
| Low | 133 (19.5) | 118 (34.0) | 180 (26.3) | 75 (21.7) |
| Missing | 4 | 1 | 1 | 3 |
| Social class (N, %) |  |  |  |  |
| High | 335 (49.1) | 126 (37.8) | 307 (46.7) | 175 (50.7) |
| Middle | 279 (40.9) | 152 (45.6) | 289 (44.0) | 144 (41.7) |
| Low | 68 (10.0) | 55 (16.5) | 61 0(9.3) | 26 0(7.5) |
| Missing | 4 | 15 | 29 | 3 |
| NS-SEC (N, %) |  |  |  |  |
| High | 340 (49.9) | 105 (31.5) | 253 (38.5) | 169 (49.0) |
| Middle | 165 (24.2) | 128 (38.4) | 259 (39.4) | 91 (26.4) |
| Low | 177 (26.0) | 100 (30.0) | 145 (22.1) | 85 (24.6) |
| Missing | 4 | 15 | 29 | 3 |
| Social comparison (median, IQR)  Missing | 4.0 (1.0)  26 | 3.0 (1.0)  13 | 3.0 (1.0)  9 | 4.0 (1.0)  6 |
| Societal ladder (median, IQR)  Missing | 7.0 (2.0)  16 | 7.0 (3.0)  4 | 7.0 (1.0)  25 | 7.0 (2.0)  17 |
| Community ladder (median, IQR)  Missing | 6.0 (3.0)  36 | 6.0 (3.0)  17 | 6.0 (2.0)  37 | 6.0 (3.0)  17 |

*Notes*: NS-SEC, National Statistics Socio-Economic Classification; PwD, person with dementia.

**Table S8: Dyadic relationship between objective social status and well-being (unadjusted)**

|  | Person with dementia social class | | Spouse caregiver social class | |
| --- | --- | --- | --- | --- |
|  | *Pp* | *pc* | *cc* | *cp* |
| Measures | Estimate (95% CI) | Estimate (95% CI) | Estimate (95% CI) | Estimate (95% CI) |

| Male PwD and female spouse caregivers (N=686) | | |  |  |
| --- | --- | --- | --- | --- |
| Education |  |  |  |  |
| High | (ref.) | (ref.) | (ref.) | (ref.) |
| Middle | 2.67 (-1.60, 6.94) | -1.76 (-6.01, 2.50) | -2.60 (-6.24, 1.04) | -0.15 (-3.77, 3.48) |
| Low | -2.70 (-6.66, 1.27) | -0.90 (-4.86, 3.06) | -4.51 (-8.24, -0.79) | -0.90 (-4.64, 2.84) |
| Social class |  |  |  |  |
| High | (ref.) | (ref.) | (ref.) | (ref.) |
| Middle | -0.43 (-3.72, 2.85) | -2.55 (-5.83, 0.73) | -2.51 (-5.76, 0.74) | 0.97 (-2.38, 4.32) |
| Low | -3.97 (-9.21, 1.27) | -0.64 (-5.88, 4.60) | -4.14 (-9.67, 1.40) | -4.43 (-9.94, 1.07) |
| NS-SEC^1^ |  |  |  |  |
| High | (ref.) | (ref.) | (ref.) | (ref.) |
| Middle | -0.70 (-4.51, 3.11) | -2.85 (-6.65, 0.94) | -0.50 (-3.99, 2.99) | 0.58 (-2.90, 4.07) |
| Low | -0.07 (-3.89, 3.75) | -0.50 (-4.34, 3.33) | -3.87 (-8.26, 0.53) | -2.48 (-6.75, 1.78) |

| Female PwD and male spouse caregivers (N=348) | | | | |
| --- | --- | --- | --- | --- |
| Education |  |  |  |  |
| High | (ref.) | (ref.) | (ref.) | (ref.) |
| Middle | -0.21 (-6.06, 5.64) | -0.08 (-5.07, 4.94) | -2.36 (-7.88, 1.41) | -2.63 (-9.15, 3.90) |
| Low | 0.63 (-4.80, 6.06) | -3.24 (-7.88, 1.41) | -7.14 (-12.14, -2.15) | -8.53 (-14.36, -2.70) |
| Social class |  |  |  |  |
| High | (ref.) | (ref.) | (ref.) | (ref.) |
| Middle | 0.28 (-5.11, 5.67) | -1.92 (-6.43, 2.59) | -1.62 (-5.87, 2.63) | -4.10 (-9.03, 0.83) |
| Low | -6.86 (-13.92, 0.19) | -4.29 (-10.36, 1.77) | -3.72 (-11.44, 4.00) | -4.22 (-13.33, 4.90) |
| NS-SEC |  |  |  |  |
| High | (ref.) | (ref.) | (ref.) | (ref.) |
| Middle | -1.58 (-7.42, 4.26) | -2.96 (-7.74, 1.82) | -2.63 (-7.44, 2.18) | -2.62 (-8.23, 2.99) |
| Low | -5.06 (-11.29, 1.17) | -4.72 (-10.00, 0.56) | -3.35 (-8.44, 1.74) | -5.21 (-11.09, 0.68) |

*Notes*: NS-SEC, National Statistics Socio-Economic Classification; PwD, people with dementia; ref, reference category; CI, confidence intervals *pp*, *pc*, *cc* and *cp* refer to the dyadic pathways shown in Figure 1.

**Table S9: Dyadic relationship between objective social status and well-being in people with dementia and their spouse caregivers (unadjusted)**

|  | Person with dementia |  | Spouse caregiver | |
| --- | --- | --- | --- | --- |
|  | *pp* | *pc* | *cc* | *cp* |
| Measures | Estimate (95% CI) | Estimate (95% CI) | Estimate (95% CI) | Estimate (95% CI) |
| Male PwD and female spouse caregivers (N=686) | | | | |
| Social comparison | 5.63 (4.20, 7.06) | 0.12 (-1.32, 1.56) | 7.27 (5.78, 8.76) | 2.52 (1.02, 4.03) |
| Social ladder | 2.54 (1.61, 3.47) | 0.48 (-0.45, 1.40) | 3.39 (2.29, 4.48) | 0.76 (-0.33, 1.85) |
| Community ladder | 2.11 (1.31, 2.91) | 0.71 (-0.09, 1.50) | 2.42 (1.56, 3.28) | 0.60 (-0.28, 1.47) |
| Female PwD and male spouse caregivers (N=348) | | | | |
| Social comparison | 8.75 (6.55, 10.94) | 1.43 (-0.56, 3.41) | 5.44 (3.28, 7.60) | 2.87 (0.58, 5.15) |
| Social ladder | 2.89 (1.49, 4.28) | 1.33 (0.09, 2.56) | 2.37 (1.00, 3.75) | 2.12 (0.56, 3.67) |
| Community ladder | 2.73 (1.58, 3.87) | 0.47 (-0.58, 1.52) | 2.46 (1.33, 3.59) | 1.66 (0.37, 2.95) |

*Note*: PwD, people with dementia; CI, confidence intervals. *pp*, *pc*, *cc* and *cp* refer to the dyadic pathways shown in Figure 1.
